# Severe *Legionella pneumonia* mimicking immune-related pneumonitis after chemoimmunotherapy for lung cancer: a case report

**DOI:** 10.3389/fonc.2025.1663978

**Published:** 2025-09-16

**Authors:** Haixian Liu, Zhaolei Ding, Lina Xu, Tao Guo

**Affiliations:** Center of Respiratory Internal Medicine Center, Weifang People’s Hospital, Weifang, Shandong, China

**Keywords:** *Legionella pneumonia*, metagenomic next-generation sequencing (tNGS), co-infection, quinolones, immune checkpoint inhibitors

## Abstract

**Background:**

Immune checkpoint inhibitors (ICIs) have significantly improved survival outcomes and quality of life in patients with various malignancies. Nevertheless, their associated toxicities must not be overlooked. Although not the most common immune-related adverse event (irAE), CIP is recognized as one of the most serious. In particular, grade 3–4 CIP that is not promptly treated may compromise subsequent immunotherapy and can result in respiratory failure or even death. *Legionnaires*’ disease, caused by *Legionella pneumophila*, is an uncommon but potentially life-threatening form of atypical pneumonia. With the expanding use of ICIs, especially in combination with chemotherapy, early stage CIP and *Legionella pneumonia* may share similar radiological features, such as ground-glass opacities, which makes early differential diagnosis difficult. However, timely differentiation is critical because the management strategies differ substantially: CIP requires systemic corticosteroids, whereas Legionella pneumonia necessitates quinolone antibiotics. Traditional diagnostic methods for *Legionella* infection, including culture on specialized media and urine antigen testing, are limited by low sensitivity and the risk of false-negative results. In recent years, targeted next-generation sequencing (tNGS) has emerged as a valuable diagnostic tool. Compared with metagenomic next-generation sequencing (mNGS), tNGS offers a shorter turnaround time, higher sensitivity and specificity, and greater cost-effectiveness. As such, it is becoming increasingly important in the accurate identification of atypical pathogens in pulmonary infections.

**Case summary:**

We report the case of a patient with squamous cell lung cancer who developed severe pneumonia following combined chemotherapy and immunotherapy. The initial working diagnosis was immune checkpoint inhibitor-related pneumonia (ICI-P) complicated by bacterial infection. However, sputum-targeted next-generation sequencing (tNGS) subsequently identified *Legionella pneumophila* infection. Following the administration of quinolone-sensitive antibiotics, the patient’s clinical condition improved markedly, and he was discharged in a stable state. A 70-year-old male farmer with a history of lung cancer, type 2 diabetes, and chronic obstructive pulmonary disease (COPD) was admitted on February 4, 2025,with fever, cough, and dyspnea following chemoimmunotherapy. He had received paclitaxel, cisplatin, and tislelizumab on January 24.Initial tests revealed leukopenia, neutropenia, and chemotherapy-induced myelosuppression. On admission, the patient exhibited hypoxemia, hyponatremia, and elevated inflammatory markers, raising suspicion for ICI-P complicated by bacterial infection. Despite empirical broad-spectrum antibiotics and corticosteroids, his condition deteriorated, requiring transfer to the Respiratory Intensive Care Unit (RICU). On February 13, tNGS of sputum identified *Legionella pneumophila*, Enterococcus faecium, *Epstein-Barr virus (EBV)*,and *Herpesvirus-1 (HSV-1).* The high relative abundance of *Legionella pneumophila* indicated it was the primary pathogen; *EBV* and *HSV-1* were presumed latent. Antimicrobial therapy was adjusted to moxifloxacin, cefepime, and ganciclovir, leading to clinical improvement and resolution of hypoxemia. Follow-up chest CT showed partial resolution of pulmonary infiltrates. The patient was discharged with home oxygen and outpatient follow-up.The patient is currently undergoing regular anti-tumor treatment.

**Conclusions:**

In the era of chemoimmunotherapy, the presence of pulmonary ground-glass interstitial lesions should prompt consideration not only of immune checkpoint inhibitor-related pneumonia (ICI-P) but also of infections caused by uncommon pathogens such as *Legionella*, particularly when there is no significant improvement after corticosteroid therapy. It is necessary to consider applying advanced molecular diagnostic techniques such as targeted next-generation sequencing (tNGS) as early as possible to make a clear diagnosis of the pathogen and guide individualized treatment.

## Introduction

In recent years, immune checkpoint inhibitors (ICIs) have brought significant advancements in cancer therapy. Numerous clinical studies conducted both domestically and internationally have demonstrated that ICIs can improve the prognosis of patients with various malignancies, offering new hope in oncology. However, immune checkpoint inhibitor-related pneumonitis (CIP) has emerged as one of the most common and potentially serious immune-related adverse events. Among patients with non-small cell lung cancer (NSCLC), the overall incidence and severity of CIP are notably higher compared to those with other tumor types. Immune checkpoint inhibitor-related pneumonia (CIP) has been reported in 2.6% to 33% of patients (1–5).

The clinical diagnosis of CIP remains challenging due to the absence of definitive mechanisms or molecular diagnostic criteria. Typically, patients without evidence of infection present with new pulmonary infiltrates on chest imaging, accompanied by dyspnea and/or other respiratory symptoms (6). Nobashi et al (7). reported that 89% of lung cancer patients with CIP exhibited ground-glass opacities on CT scans. They also observed features such as organizing pneumonia and caseous pneumonia. However, these radiological findings are non-specific and overlap with a broad spectrum of pulmonary conditions, including infectious pneumonias and bacterial lung abscesses. Immunotherapy is often administered in combination with chemotherapy. However, chemotherapy-induced bone marrow suppression increases the risk of infection and complicates the diagnostic process for interstitial lung disease following combined treatment especially when radiologic findings are atypical and the causative pathogen is unclear.

*Legionella pneumophila* is a well-recognized pathogen responsible for severe community-acquired pneumonia (CAP) and an increasingly important opportunistic agent in hospital-acquired infections, particularly in immunocompromised individuals (8).*Legionnaires*’ disease, although relatively rare, carries a high mortality rate and primarily affects elderly patients and those with underlying chronic pulmonary diseases or immunosuppression (9).Traditional diagnostic modalities—such as urinary antigen testing and culture—remain essential tools for detecting *L. pneumophila*, yet their limitations (e.g., low sensitivity for non–serogroup 1 strains and slow turnaround time) frequently hinder timely and targeted treatment (10).*Legionella pneumonia* is known for its rapid progression, with approximately 10% to 30% of severe cases developing into acute respiratory distress syndrome (ARDS), necessitating mechanical ventilation or extracorporeal membrane oxygenation (ECMO) support. These invasive interventions, along with the use of broad-spectrum antibiotics and immunosuppressive agents such as glucocorticoids, create an environment conducive to secondary infections. The diagnostic complexity increases significantly in oncology patients receiving immune checkpoint inhibitors (ICIs), as pulmonary complications particularly immune checkpoint inhibitor-associated pneumonitis (ICI-P) can clinically and radiographically mimic infectious pneumonia (11). This overlap underscores the urgent need for rapid and accurate pathogen identification to prevent inappropriate corticosteroid administration or delays in targeted antimicrobial therapy. While macrolides and respiratory fluoroquinolones remain the first-line treatments for *Legionella pneumonia*, the presence of co-infections with multidrug-resistant bacteria or viruses especially in immunocompromised hosts further complicates empirical treatment strategies (12).Recent advancements in metagenomic next-generation sequencing (mNGS), including targeted NGS (tNGS), have shown great promise in revolutionizing pathogen detection by enabling unbiased identification of microbial nucleic acids directly from clinical samples (13).However, its utility in differentiating ICI-P from atypical infections such as Legionella pneumonia remains underreported, particularly in resource-constrained healthcare settings.

Here, we report a case of a patient with locally advanced lung cancer who developed *Legionella pneumonia* during chemotherapy and immunotherapy and was initially misdiagnosed with ICI-P. Using tNGS-based diagnostics, the treatment regimen was rapidly optimized, leading to clinical recovery. This case highlights the critical role of integrating tNGS into the diagnostic and therapeutic workflow for severe pneumonia in high-risk, immunocompromised populations.

## Case presentation

A 70-year-old male farmer with a one-month history of biopsy-confirmed lung cancer was admitted to our department on February 4, 2025, due to a three-day history of fever. His past medical history included cerebral infarction (7 years ago, without significant sequelae), chronic hepatitis B (diagnosed 5 years ago and managed with oral tenofovir disoproxil fumarate 25 mg once daily), type 2 diabetes mellitus (diagnosed 5 years ago, controlled with glimepiride), and recently diagnosed chronic obstructive pulmonary disease (COPD) for about one month. The patient reported a 50-year smoking history (approximately 20 cigarettes/day) and occasional alcohol consumption over the past 40 years. He had received one cycle of combination chemotherapy and immunotherapy (paclitaxel, cisplatin, and tislelizumab) on January 24, 2025. Physical examination on admission: The patient’s body temperature was 37.8 °C, pulse 88 beats/min, respiratory rate 19 breaths/min, and blood pressure 122/74 mmHg. Auscultation of both lungs revealed coarse breath sounds with audible wet rales, while dry rales were absent. Three days prior to admission, he developed a fever (maximum temperature 38.5 °C) accompanied by chills, a cough with scant white sputum, and mild exertional dyspnea. There were no reports of hemoptysis, night sweats, or fatigue. Initial laboratory investigations at a local hospital on February 4 revealed leukopenia (WBC 1.85×10^9^/L) and neutropenia (GRAN 0.8×10^9^/L),suggestive of chemotherapy-induced myelosuppression. Subcutaneous recombinant human granulocyte colony-stimulating factor (0.3 mg) was administered. In light of the recent administration of tislelizumab, along with bone marrow suppression and elevated inflammatory markers. The patient was over 65 years of age, had a long history of smoking, chronic obstructive pulmonary disease, and lung squamous cell carcinoma. He was treated with the PD-1 monoclonal antibody tislelizumab. At the time of admission, chest CT performed outside the hospital revealed multiple ground-glass opacities. Considering the patient’s medical history in combination with the available auxiliary examinations, the condition was preliminarily considered to be immune checkpoint inhibitor-related pneumonia (ICI-P) complicated by bacterial infection. On admission, arterial blood gas analysis revealed pH 7.48, PaCO2 33.2 mmHg, PaO2 63.5 mmHg, and oxygen saturation (SpO2) 92.7%, indicating hypoxemia. Serum sodium was markedly decreased (Na^+^ 117 mmol/L). On February 5, laboratory tests showed leukocytosis (WBC 13.79 × 10^9^/L), neutrophilia (GRAN 95.8%), and elevated C-reactive protein (CRP 187.30 mg/L). Empirical treatment was initiated with piperacillin-tazobactam (4.5 g every 8 hours) and methylprednisolone (40 mg twice daily) for suspected ICI-P. However, the patient’s symptoms progressed, with worsening dyspnea, persistent fever, and rising CRP levels.

Methylprednisolone was escalated to 80 mg twice daily. Antimicrobial therapy was broadened to include meropenem (1 g every 8 hours), oral voriconazole (200 mg every 12 hours), and intravenous immunoglobulin. Despite high-flow oxygen therapy, hypoxemia persisted (PaO2 57.3 mmHg, SpO2 88.3%). Given the patient’s immunocompromised status and the possibility of concomitant bacterial infection, vancomycin (500 mg every 8 hours) was added to the treatment regimen, and non-invasive ventilation was initiated. Due to progressive respiratory failure and the development of urinary incontinence, the patient was transferred to the Respiratory Intensive Care Unit (RICU). Despite the use of high-dose corticosteroids and broad-spectrum antibiotics targeting Gram-negative bacilli, Gram-positive cocci, and fungi (including piperacillin-tazobactam, meropenem, voriconazole, and vancomycin),the patient showed no significant clinical improvement. The broad-spectrum antimicrobial therapy and glucocorticoid anti-inflammatory treatment demonstrated poor efficacy. At this stage, there was increasing concern that the condition was not merely immune checkpoint inhibitor-related pneumonia (ICI-P) combined with a common bacterial infection. The antibiotics and treatment regimen in use did not appear to adequately cover the causative pathogen.

On February 13, sputum-targeted next-generation sequencing (tNGS, Shilu Medical) identified *Legionella pneumophila* (65,431 sequence reads), *Enterococcus faecalis* (29,237 reads), *Epstein–Barr virus* (EBV; 13,678 reads), and *Human herpesvirus 1* (HSV-1; 858 reads) (**Table 1**). Clinically, the patient exhibited acute respiratory symptoms, high-grade fever, bilateral pulmonary infiltrates, and rapidly progressing interstitial changes—findings consistent with *Legionella pneumophila*. Although tNGS can detect multiple pathogens, clinical interpretation depends on relative abundance and pathogenicity. The read counts and relative abundance of *Legionella pneumophila* were significantly higher than those of other pathogens, supporting its role as the primary causative agent. Urinary antigen testing (UAT) for *Legionella* was not performed in this case, as the patient’s critical condition and the limited sensitivity of UAT for non–serogroup 1 strains rendered this method less suitable. Instead, targeted next-generation sequencing (tNGS) of sputum was performed and rapidly identified *Legionella pneumophila*, which established the diagnosis. Furthermore, *EBV* and *HSV-1* were also detected by tNGS. *Epstein–Barr virus (EBV)* is a ubiquitous pathogen, with more than 90% of adults worldwide being carriers. The *HSV-1* sequence count was relatively low, and both viruses are generally regarded as latent viruses that may reactivate under certain conditions. Although their direct clinical relevance in this case was limited, the patient’s immunocompromised state suggested a tendency toward accelerated viral replication. Therefore, ganciclovir was initiated to target *EBV*, recognizing that while it is not the first-line agent for *HSV-*1, it may still exert partial antiviral activity. *Enterococcus faecium* was the second most abundant pathogen detected by sequence count. Although it is a common commensal organism in the human gastrointestinal tract, its detection in sputum could represent colonization or contamination. However, given the relatively high read number and the patient’s compromised immune status, coverage against *E. faecium* was considered necessary. The patient had previously received vancomycin without clinical benefit, and sputum culture with susceptibility testing could not be obtained. A domestic case report from China documented successful treatment of *E. faecium* sepsis with cefobenzoate; therefore, cefobenzoate was added to the treatment regimen.

**Table 1:** tNGS result (sputum).

Pathogen Detection Results (Main report)				
type	Strain Designation	normalized sequence counts	estimated pathogen concentration(copies/ml)	
G-	Legionella_pneumophila	65431	2×10^5^	
G+	Enterococcus_faecium	29237	1×10^5^	
DNA virus	Epstrin-Barr_virus	13678	5×10^4^	
DNA virus	Herpesvirus_1	858	3×10^3^	
Pathogen detection results (background flora)				
type	Strain Designation	normalized sequence counts		estimated pathogen concentration(copies/ml)
G+	Staphylococcus_hominis	16241		7×10^4^
G+	Staphylococcus_epidermidis	9096		3×10^4^
DNA virus	TTV-like_mini_virus	116		6×10^2^
Fungus	Saccharomyces_cerevisiae	69		3×10^2^
G-	Vibro_parahaemolytius	7		<1×10^2^
Gray zone				
type	Strain Designation	normalized sequence counts		estimated pathogen concentration(copies/ml)
Fungus	Fusarium_verticilioides	1		<1×10^2^

**Figure 1:**
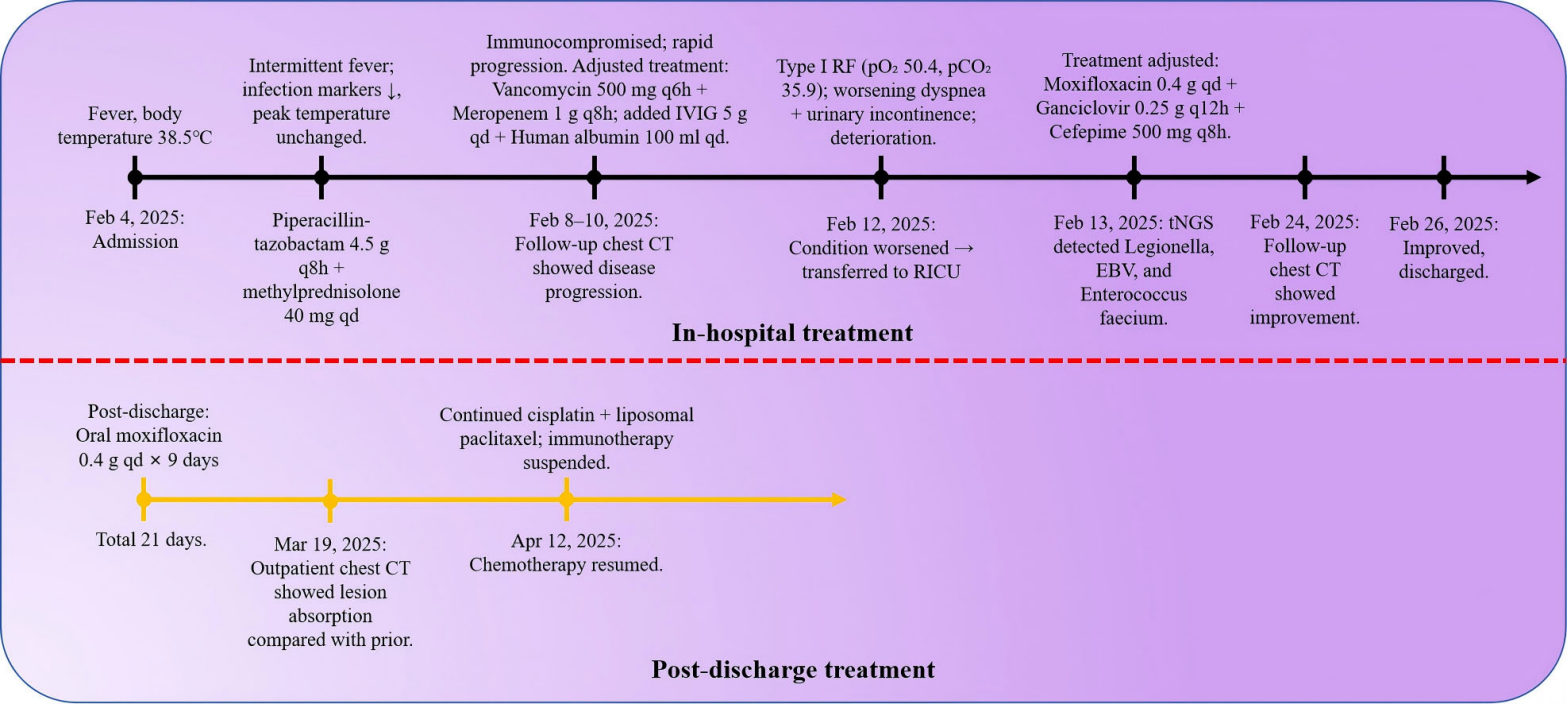
Major changes in the condition and adjustments to the treatment plan.

**Figure 2:**
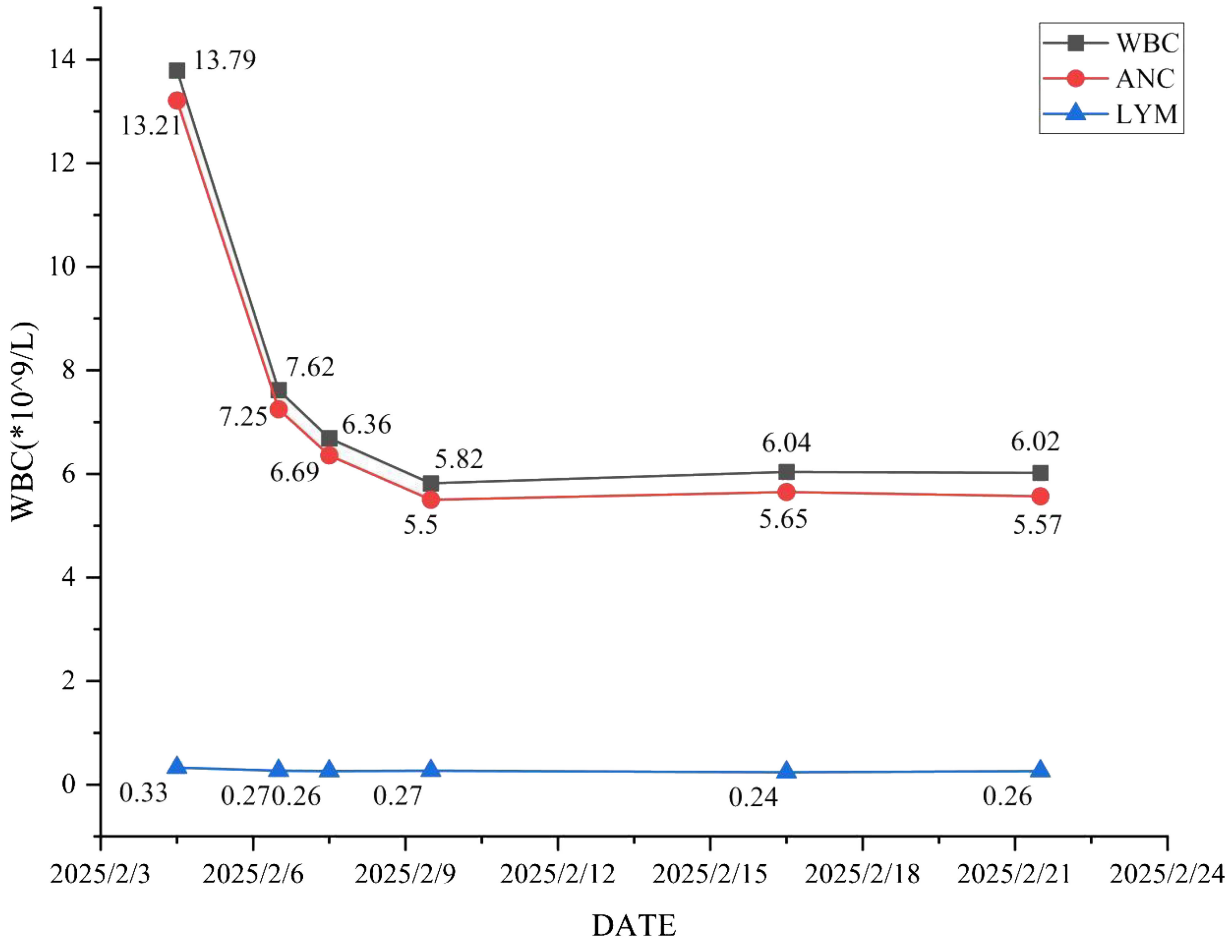
The changing trends of white blood cell count, absolute neutrophil count and absolute lymphocyte count.

**Figure 3:**
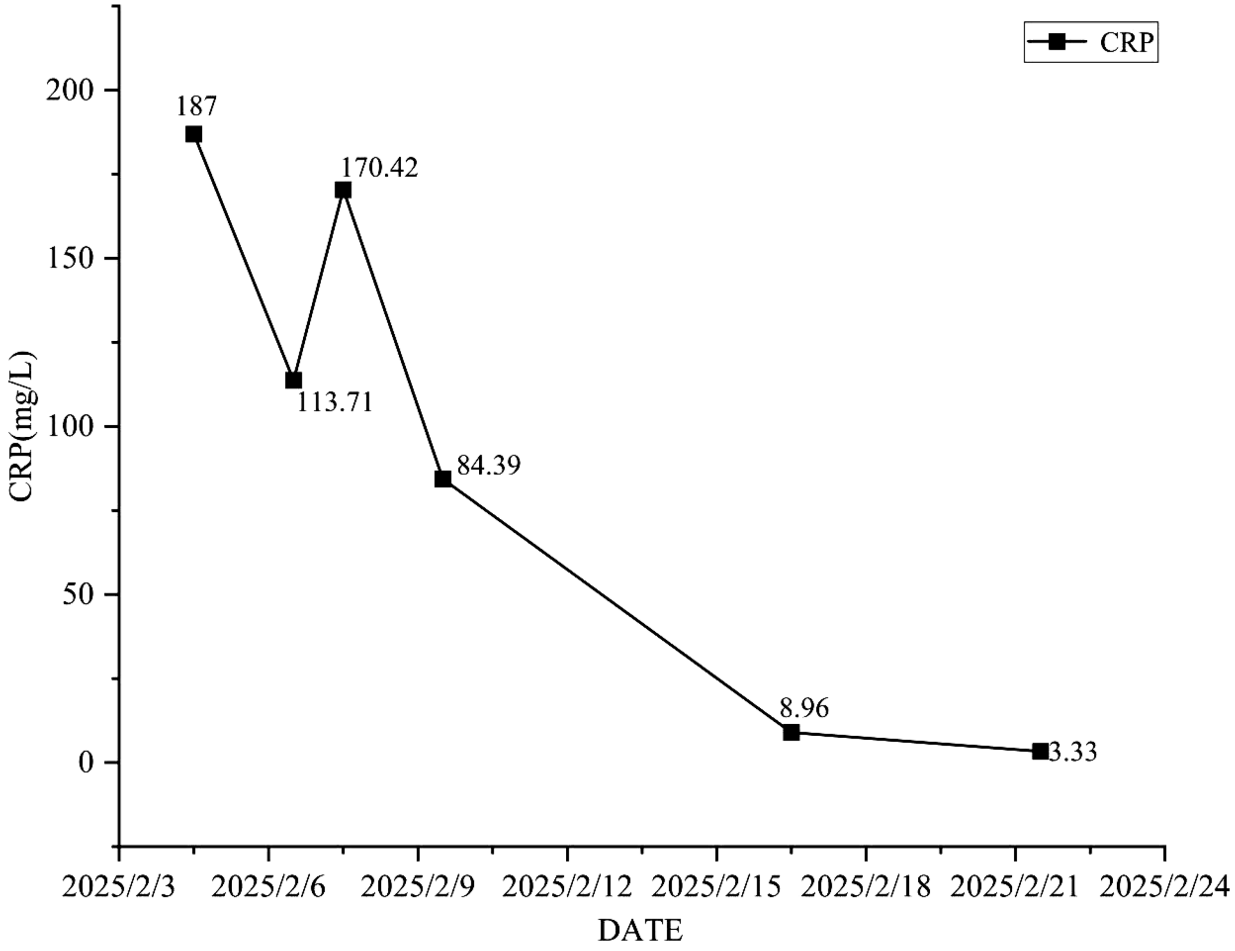
The changing trend of C-reactive protein.

**Figure 4:**
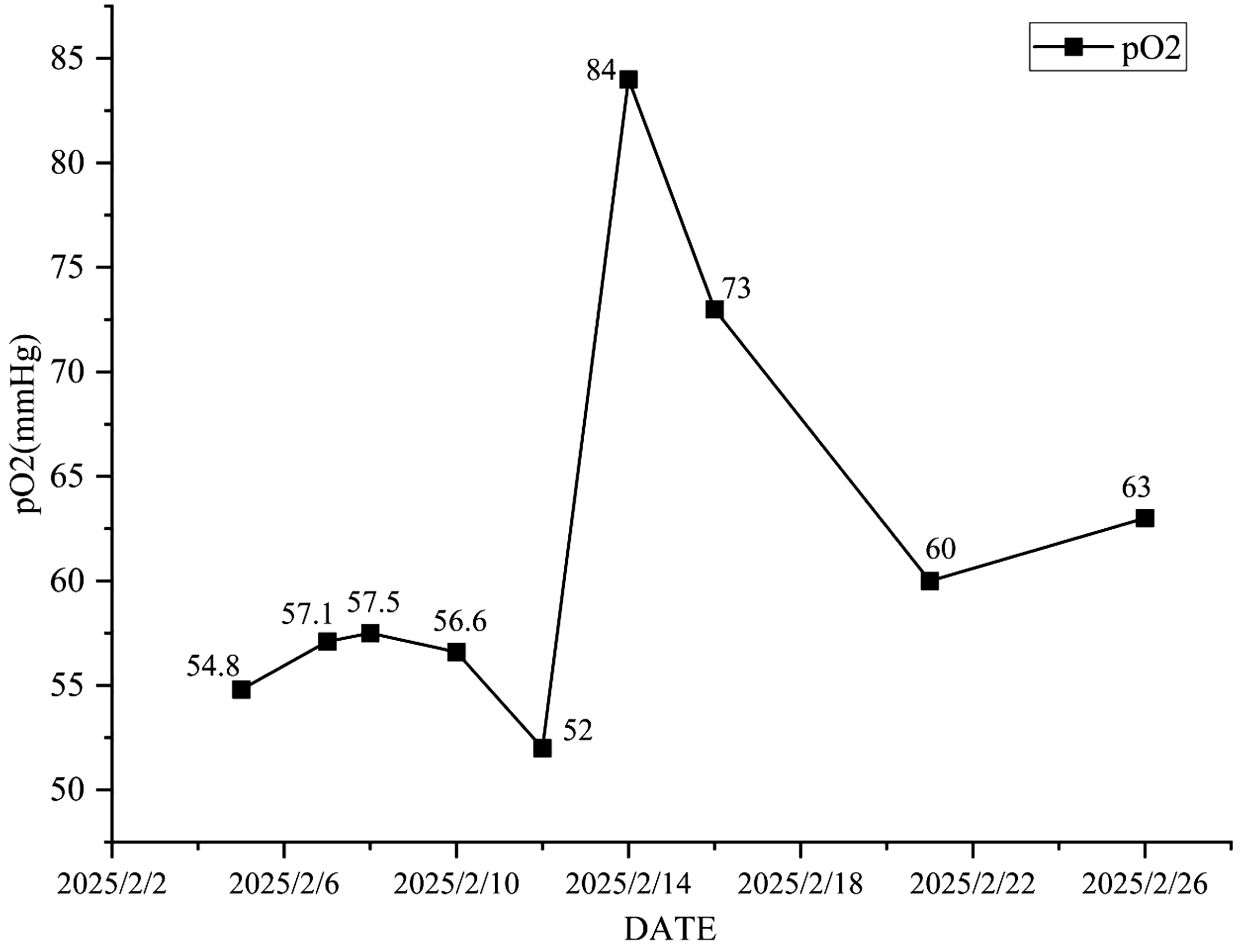
The changing trend of arterial partial pressure of oxygen.

**Figure 5:**
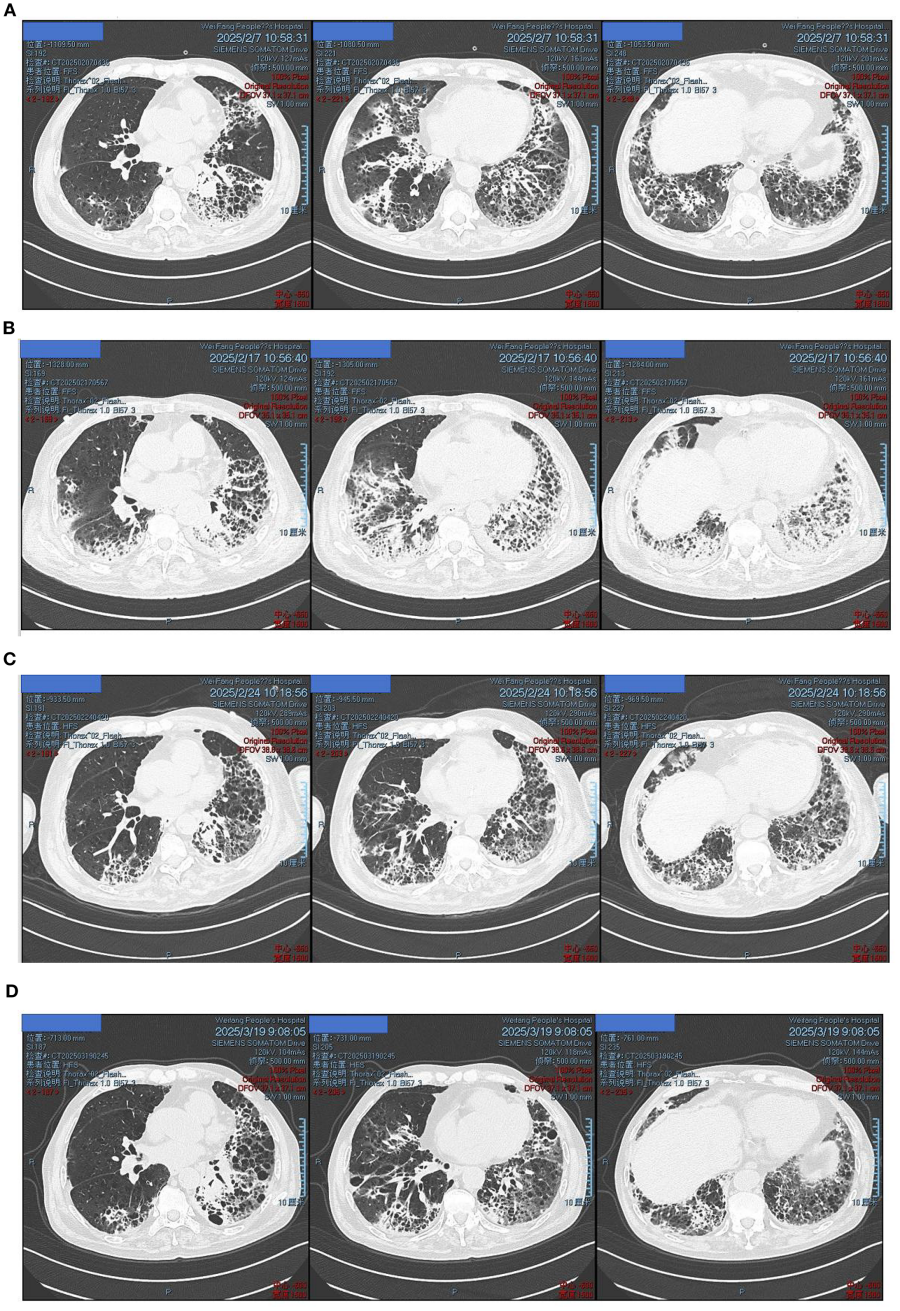
The changes of the patient’s chest CT. **(A)** The patient had repeated fever after admission. On February 7, 2025, the chest CT was reexamined, indicating that the condition was more severe than before admission. **(B)** Due to the aggravation of the patient’s chest tightness and the continuous progression of the condition, a chest CT re-examination was conducted on February 17, 2025, indicating the aggravation of pneumonia. **(C)** One week after switching to the sensitive antibiotic, a chest CT was reexamined on February 24, 2025, and there was an improvement in absorption compared to February 17, 2025. **(D)** The chest CT of the patient was reexamined at the outpatient department 13 days after discharge, and the lesion improved further.

**Figure 6:**
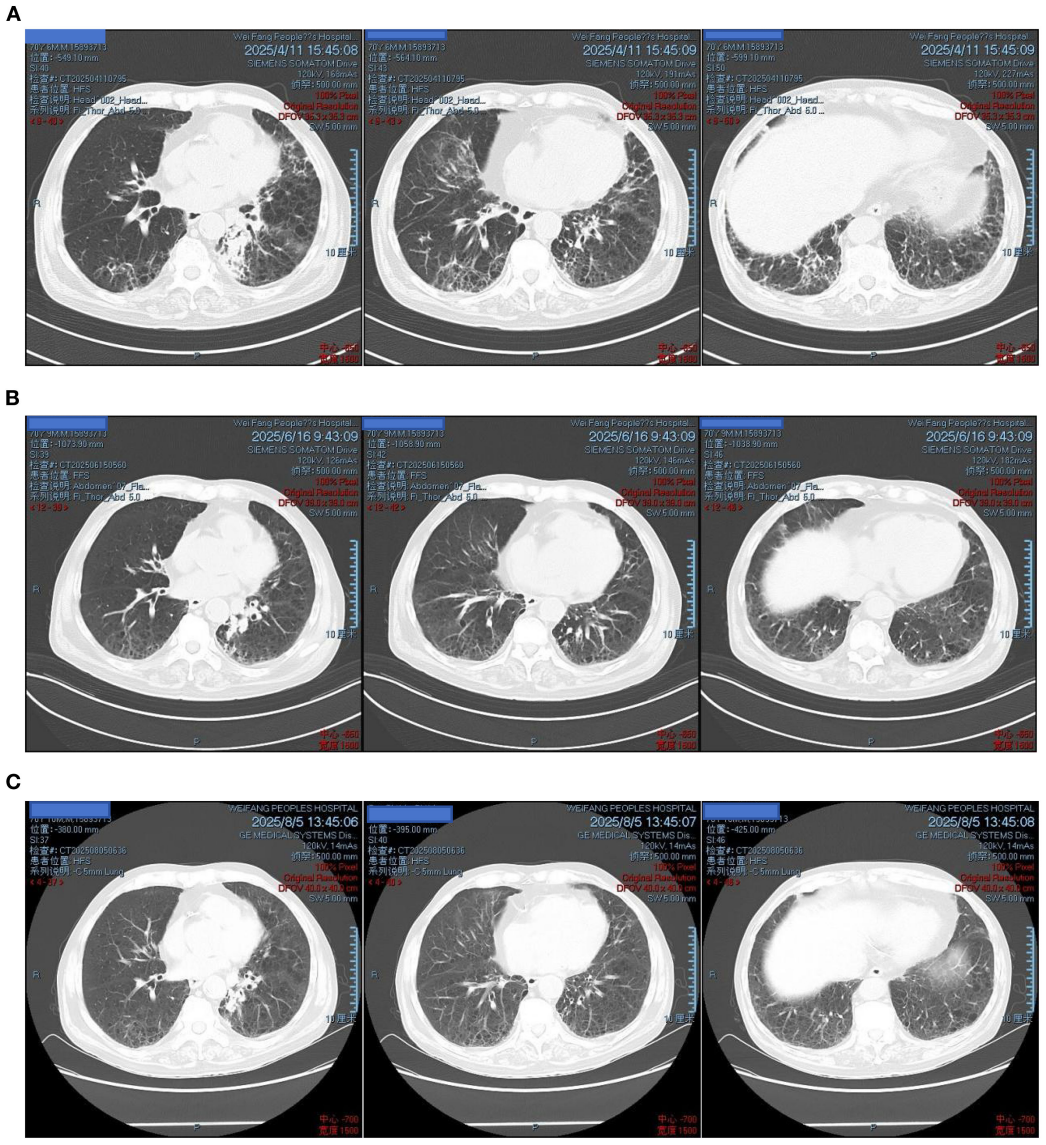
Follow-up chest CT after discharge: **(A)** April 11, 2025 – bilateral pulmonary lesions improved; **(B)** June 16, 2025 – further improvement; **(C)** August 5, 2025 – marked absorption of lesions.

Antimicrobial therapy was modified to include moxifloxacin (400 mg once daily), cefobiprole (500 mg every 8 hours), and ganciclovir (250 mg every 12 hours). Methylprednisolone was gradually tapered. The patient showed marked clinical improvement, with resolution of hypoxemia (oxygen saturation improved to 96% on 60% high-flow oxygen) (**Figure 1**). In addition, the infection indicators have significantly decreased (**Figures 2**–**4**). Follow-up chest CT (**Figure 5**) demonstrated partial resolution of pulmonary infiltrates. At the family’s request, the patient was discharged for continued home oxygen therapy and outpatient follow-up. The most recent chest CT (**Figure 6**), performed on August 5, 2025, demonstrated significant resolution of the *Legionella* pneumonia. The patient has since resumed and is continuing a regular course of anti-tumor therapy.

## Discussion

Immune checkpoint inhibitors (ICIs) have markedly improved the prognosis of patients with various malignancies; however, immune-related adverse events (irAEs) remain a major clinical challenge. Among them, immune checkpoint inhibitor-related pneumonia (CIP) is one of the most severe complications, often necessitating treatment discontinuation and even carrying a risk of mortality. In the present case, a 70-year-old patient with lung cancer developed severe pneumonia following chemoimmunotherapy, which was initially suspected to be CIP. However, further diagnostic evaluation confirmed *Legionella pneumophila* infection, highlighting the importance of considering opportunistic pathogens in immunocompromised patients receiving ICIs.

*Legionella* is an aerobic, Gram-negative γ-proteobacterium (14). To date, over 50 species and more than 60 serogroups of Legionella have been identified, with at least 24 species known to cause lower respiratory tract infections in humans (15). Approximately 90% of *Legionella pneumonia* cases are attributed to *Legionella pneumophila serogroup 1* (LP1), which thrives in warm, humid environments and replicates efficiently in water at 25 – 42 °C, with an optimal growth temperature of 35 – 37 °C (16, 17). Humans are typically infected by inhaling aerosolized droplets contaminated from artificial water systems, such as showers, plumbing, and air conditioning units (9). Once inhaled, Legionella invades alveolar macrophages via flagella, pili, and outer membrane components, proliferating within vacuoles. The bacterium ultimately induces apoptosis through caspase-1 activation, triggering recurrent replication cycles and culminating in *Legionella pneumonia (* 18–20).This 70-year-old patient had a history of smoking and chronic obstructive pulmonary disease. Prior to disease onset, he had received chemotherapy combined with immunotherapy. The chemotherapy induced bone marrow suppression, resulting in impaired immune function and predisposing him to *Legionella* infection. The infection progressed rapidly, leading to respiratory failure.

Despite its environmental ubiquity, clinical infection remains uncommon. Known risk factors include age >50 years, male sex, smoking, alcohol abuse, immunosuppression, and pre-existing cardiopulmonary diseases (21–23).Delayed diagnosis and treatment, coupled with these comorbidities, are associated with increased mortality (24).Because the primary host defense mechanism against *Legionella* is cell-mediated immunity, individuals with impaired immune function including those with diabetes, malignancies, AIDS, or on immunosuppressive therapies (e.g., corticosteroids, TNF- α inhibitors) are at significantly elevated risk (5, 21, 23, 25, 26).

Immune checkpoint inhibitor-related pneumonitis (CIP) is a relatively common and potentially life-threatening immune-related adverse event (irAE) in lung cancer patients undergoing immunotherapy. The incidence of CIP in non-small cell lung cancer (NSCLC) patients is estimated at 3. 1%, significantly higher than in other malignancies such as melanoma or urothelial carcinoma (2.0%) (24). Risk factors include male sex, age ≥ 65 years, smoking history, squamous cell histology, pre-existing lung disease, and concurrent systemic therapy (24, 25). CIP typically occurs within 2–3 months of initiating immunotherapy, with lung cancer patients demonstrating earlier onset (median: 1.1 months) than those with other tumor types (3. 1 months) (5, 26, 27).

In this case, an elderly male with squamous cell lung carcinoma developed fever and interstitial lung changes after receiving chemoimmunotherapy (paclitaxel, cisplatin, and tislelizumab). He had a substantial smoking history and comorbid COPD. Initial imaging revealed diffuse interstitial infiltrates without focal consolidation. Given his recent exposure to tislelizumab, concurrent bone marrow suppression, and elevated inflammatory markers, immune-related pneumonitis with possible bacterial co-infection was initially suspected. Accordingly, empirical therapy with piperacillin-tazobactam and methylprednisolone was initiated. However, the patient ‘s condition worsened, with persistent fever, rising inflammatory markers, and progression to respiratory failure, necessitating reconsideration of the initial diagnosis. Considering the patient ‘ s immunocompromised state and poor response to empirical therapy, atypical pathogens were suspected. Bronchoalveolar lavage was deemed unsafe due to the patient’s critical condition. Therefore, sputum targeted next-generation sequencing (tNGS) was performed. The results revealed high relative abundance of *L.pneumophila*, confirming the diagnosis of *Legionella pneumonia. EBV* and *HSV-1* were also detected, likely due to viral reactivation rather than active infection. Enterococcus faecium, not typically a respiratory pathogen, was considered colonization or contamination. In this case, urinary antigen testing (UAT) was not performed. Although UAT remains a rapid and convenient diagnostic tool, its sensitivity is limited to *Legionella pneumophila* serogroup 1, which may lead to false-negative results in non–serogroup 1 infections. Given the patient’s rapid deterioration and critical illness, tNGS was prioritized, allowing timely identification of *Legionella pneumophila* and guiding appropriate antimicrobial therapy.

*Legionella pneumonia* is especially common in immunocompromised individuals, such as those with chronic lung disease, diabetes, malignancy, long-term corticosteroid use, and the elderly. The pathogen ‘s intracellular replication in alveolar macrophages leads to intense local inflammation, alveolar damage, and immune dysregulation. This in turn disrupts pulmonary defense mechanisms, predisposing patients to secondary infections. In this case, rapid deterioration and respiratory failure suggest coexisting or opportunistic infections, which were addressed through broad-spectrum coverage and targeted antiviral prophylaxis (ganciclovir for *EBV*).

Traditional diagnostic methods for *Legionella* include urinary antigen testing, culture, PCR, and serology. Urinary antigen testing, although rapid, has variable sensitivity (55- 80%) and is limited to detecting *Legionella pneumonia* type 1 (28). Culture remains the gold standard but requires specialized media and takes 3–5 days, limiting its utility in urgent clinical settings. Nucleic acid amplification tests (NAATs), such as PCR and sequencing, offer faster and more sensitive detection. In particular, tNGS allows broad-spectrum, unbiased identification of pathogens directly from clinical specimens and has been increasingly adopted in critical care diagnostics (29).

In this case, tNGS enabled rapid identification of *Legionella pneumophila*, guiding timely adjustment of antimicrobial therapy. The patient responded well to moxifloxacin, cefobiprole, and ganciclovir, with subsequent clinical improvement and avoidance of invasive ventilation. However, several limitations should be acknowledged. Financial constraints precluded the use of metagenomic NGS (mNGS), and follow-up tNGS was not performed, leaving uncertainty regarding pathogen clearance. The success of this patient was attributed to the precise detection of tNGS. However, if the patient had undergone tNGS testing at an earlier stage, before having any evidence of pathogen, and had received sensitive antibiotics earlier, it is possible that the progression of the patient’s condition could have been slowed down, and they might not have been admitted to the RICU. Furthermore, in patients with multiple comorbidities or those who have undergone chemotherapy or immunotherapy with immunosuppressive agents, atypical manifestations in imaging or hematological parameters may occur, increasing the risk of missed or incorrect diagnoses. Distinguishing between immune checkpoint inhibitor-related pneumonia (CIP) and infections caused by specific pathogens is therefore critical, as the therapeutic strategies differ substantially—glucocorticoid anti-inflammatory therapy for CIP versus targeted anti-infective therapy for infectious pneumonia. In the present case, sputum culture was negative, which is not unexpected given the difficulty of culturing Legionella. As the disease progressed, sputum-targeted next-generation sequencing (tNGS) was performed, which identified *Legionella pneumophila* and enabled adjustment to sensitive antibiotic therapy. This resulted in clinical recovery. The case highlights the value of tNGS in overcoming the limitations of conventional diagnostic methods, such as the suboptimal sensitivity of urine antigen detection and the prolonged turnaround time of culture. By rapidly identifying *Legionella* in sputum, tNGS facilitated timely adjustment to quinolone therapy, thereby avoiding invasive interventions and achieving favorable clinical outcomes. This underscores the importance of accurate and timely pathogen detection in the management of severe pneumonia. In addition, because sputum culture yielded no pathogenic bacteria and conventional empirical therapy proved ineffective, targeted next-generation sequencing (tNGS) was performed. The rapid identification of Legionella pneumophila was decisive for clinical management. Compared with traditional methods, tNGS offers higher sensitivity, the ability to simultaneously detect multiple pathogens, and a shorter turnaround time, making it particularly valuable in critically ill or immunocompromised patients. Nevertheless, its clinical application has several limitations. First, tNGS cannot replace conventional culture, which remains indispensable for antimicrobial susceptibility testing. Second, the high cost and limited accessibility of tNGS restrict its widespread use. Third, interpretation of sequencing results requires integration with clinical context; otherwise, commensal organisms or latent viruses may be misclassified as causative pathogens. Therefore, tNGS should not be regarded as a stand-alone diagnostic tool but rather as an important adjunct to routine testing, especially in situations where conventional methods fail to identify the etiology or when patients deteriorate rapidly under broad-spectrum treatment. In the present case, the timely application of tNGS not only confirmed the pathogen but also guided targeted antimicrobial therapy, ultimately leading to a favorable outcome.

In this case, tNGS revealed not only Legionella pneumophila but also *Enterococcus faecium*, *Epstein–Barr virus* (*EBV*), and herpes simplex virus type 1 (*HSV-1*). While the high relative abundance of *L. pneumophila* supported its role as the primary pathogen, *EBV* and *HSV-1* were considered latent viruses that may have reactivated under the patient’s immunocompromised condition, and *E. faecium* was regarded as possible colonization. Nevertheless, these findings highlight the complexity of co-infections in immunocompromised hosts, where multiple pathogens may coexist and complicate both diagnosis and treatment. Azithromycin and levofloxacin are the preferred antibiotics because they possess bactericidal properties and can achieve high intracellular concentrations. For severe legionella pneumonia, the efficacy of quinolones is superior to that of macrolides (30). Cunha notes treatment for less than 2 weeks can increase the risk of relapse (31).In our case, clinical improvement was observed shortly after the antimicrobial regimen was adjusted to include moxifloxacin, underscoring the pivotal role of fluoroquinolones in targeted therapy against *L. pneumophila*. This case further illustrates the importance of promptly considering fluoroquinolones when *Legionella* infection is suspected, particularly in critically ill patients who fail to respond to broad-spectrum empirical treatment. However, severe pneumonia, especially in patients with compromised immunity, is often not caused by a single pathogen. In this case, the patient was infected with *Enterococcus faecalis* and *Epstein-Barr virus*. Therefore, in clinical practice, it is important to continuously assess the treatment effect and adjust the treatment accordingly.

This case illustrates the diagnostic challenges in distinguishing CIP from atypical infections and underscores the clinical utility of tNGS in critically ill, immunocompromised patients. Early identification and tailored antimicrobial therapy were crucial in achieving a favorable outcome.

## Conclusions

This case highlights that in patients receiving chemotherapy combined with immunotherapy, the development of diffuse ground-glass opacities and respiratory failure warrants a high level of vigilance in differentiating immune checkpoint inhibitor-related pneumonia from rare infections caused by atypical pathogens. For patients who do not respond to glucocorticoids or conventional antimicrobial therapy, advanced molecular diagnostic techniques such as targeted next-generation sequencing (tNGS) should be promptly considered to establish an etiological diagnosis and guide individualized treatment. In this case, the application of tNGS enabled the rapid identification of *Legionella pneumophil*a and facilitated timely adjustment of the antimicrobial regimen, thereby preventing further clinical deterioration. This underscores the significant clinical value of tNGS in the diagnosis and management of complex infections, particularly in immunocompromised hosts.

## Data Availability

The raw data supporting the conclusions of this article will be made available by the authors, without undue reservation.

## References

[1] Yang L Li B Xu Y Zou B Fan B Wang C. Pneumonitis with combined immune checkpoint inhibitors and chemoradiotherapy in locally advanced non-small-cell lung cancer: a systematic review and meta-analysis. Future Oncol. (2023) 19:1151–60. doi: 10.2217/fon-2022-1274, PMID: 37293787

[2] Naidoo J Wang X Woo KM Iyriboz T Halpenny D Cunningham J. Pneumonitis in patients treated with anti-programmed death-1/programmed death ligand 1 therapy. J Clin Oncol. (2017) 35:709–17. doi: 10.1200/JCO.2016.68.2005, PMID: 27646942 PMC5559901

[3] Ma K Lu Y Jiang S Tang J Li X Zhang Y. The relative risk and incidence of immune checkpoint inhibitors related pneumonitis in patients with advanced cancer: A meta-analysis. Front Pharmacol. (2018) 9:1430. doi: 10.3389/fphar.2018.01430, PMID: 30618738 PMC6297260

[4] Atchley WT Alvarez C Saxena-Beem S Schwartz TA Ishizawar RC Patel KP. Immune checkpoint inhibitor-related pneumonitis in lung cancer: real-world incidence, risk factors, and management practices across six health care centers in north carolina. Chest. (2021) 160:731–42. doi: 10.1016/j.chest.2021.02.032, PMID: 33621599 PMC8411447

[5] Suresh K Voong KR Shankar B Forde PM Ettinger DS Marrone KA. Pneumonitis in non-small cell lung cancer patients receiving immune checkpoint immunotherapy: incidence and risk factors. J Thorac Oncol. (2018) 13:1930–9. doi: 10.1016/j.jtho.2018.08.2035, PMID: 30267842

[6] Schneider BJ Naidoo J Santomasso BD Lacchetti C Adkins S Anadkat M. Management of immune-related adverse events in patients treated with immune checkpoint inhibitor therapy: ASCO guideline update. J Clin Oncol. (2021) 39:4073–126. doi: 10.1200/JCO.21.01440, PMID: 34724392

[7] Nobashi TW Nishimoto Y Kawata Y Yutani H Nakamura M Tsuji Y. Clinical and radiological features of immune checkpoint inhibitor-related pneumonitis in lung cancer and non-lung cancers. Br J Radiol. (2020) 93:20200409. doi: 10.1259/bjr.20200409, PMID: 32783627 PMC8519648

[8] Chahin A Opal SM. Severe pneumonia caused by legionella pneumophila: differential diagnosis and therapeutic considerations. Infect Dis Clin North Am. (2017) 31:111–21. doi: 10.1016/j.idc.2016.10.009, PMID: 28159171 PMC7135102

[9] Yang Z Yang W. Severe cavitary pneumonia caused by legionella pneumophila: a case report. Clin Lab. (2023) 69:1509–12. doi: 10.7754/Clin.Lab.2022.221114, PMID: 37436399

[10] Viasus D Gaia V Manzur-Barbur C Carratalà J. Legionnaires’ Disease: update on diagnosis and treatment. Infect Dis Ther. (2022) 11:973–86. doi: 10.1007/s40121-022-00635-7, PMID: 35505000 PMC9124264

[11] Trousil J Frgelecová L Kubíčková P Řeháková K Drašar V Matějková J. Acute pneumonia caused by clinically isolated legionella pneumophila sg 1, ST 62: host responses and pathologies in mice. Microorganisms. (2022) 10:179. doi: 10.3390/microorganisms10010179, PMID: 35056629 PMC8781576

[12] Lu Z Zhang A Guo J Ni H. An unusual case of severe pneumonia caused by Tropheryma whipplei combined with Legionella pneumophila. World J Emerg Med. (2023) 14:492–4. doi: 10.5847/wjem.j.1920-8642.2023.095, PMID: 37969216 PMC10632750

[13] Yin Y Butler C Zhang Q. Challenges in the application of NGS in the clinical laboratory. Hum Immunol. (2021) 82:812–9. doi: 10.1016/j.humimm.2021.03.011, PMID: 33892986

[14] Mondino S Schmidt S Rolando M Escoll P Gomez-Valero L Buchrieser C. Legionnaires’ Disease: state of the art knowledge of pathogenesis mechanisms of legionella. Annu Rev Pathol. (2020) 15:439–66. doi: 10.1146/annurev-pathmechdis-012419-032742, PMID: 31657966

[15] Yu VL Plouffe JF Pastoris MC Stout JE Schousboe M Widmer A. Distribution of Legionella species and serogroups isolated by culture in patients with sporadic community-acquired legionellosis: an international collaborative survey. J Infect Dis. (2002) 186:127–8. doi: 10.1086/341087, PMID: 12089674

[16] Jericó Alba C Nogués Solán X Santos Martínez MJ Félez Flor M Garcés Jarque JM Mariñosa Marré M. Brote epidémico de neumonía comunitaria por Legionella pneumophila en Barcelona: “el brote de la Barceloneta”. Efecto del diagnóstico y tratamiento precoz [Legionella pneumophila pneumonia community epidemic outbreak in Barcelona: “The Barceloneta outbreak”. Effect on the early diagnosis and treatment. Rev Clin Esp. (2004) 204:70–4. doi: 10.1157/13058800, PMID: 15023304

[17] van Heijnsbergen E de Roda Husman AM Lodder WJ Bouwknegt M Docters van Leeuwen AE Bruin JP. Viable Legionella pneumophila bacteria in natural soil and rainwater puddles. J Appl Microbiol. (2014) 117:882–90. doi: 10.1111/jam.12559, PMID: 24888231

[18] Newton HJ Ang DK van Driel IR Hartland EL. Molecular pathogenesis of infections caused by Legionella pneumophila. Clin Microbiol Rev. (2010) 23:274–98. doi: 10.1128/CMR.00052-09, PMID: 20375353 PMC2863363

[19] Hammer BK Tateda ES Swanson MS. A two-component regulator induces the transmission phenotype of stationary-phase Legionella pneumophila. Mol Microbiol. (2002) 44:107–18. doi: 10.1046/j.1365-2958.2002.02884.x, PMID: 11967072 PMC13220096

[20] Gao LY Abu Kwaik Y. Apoptosis in macrophages and alveolar epithelial cells during early stages of infection by Legionella pneumophila and its role in cytopathogenicity. Infect Immun. (1999) 67:862–70. doi: 10.1128/IAI.67.2.862-870.1999, PMID: 9916101 PMC96397

[21] Carratala J Gudiol F Pallares R Dorca J Verdaguer R Ariza J. Risk factors for nosocomial Legionella pneumophila pneumonia. Am J Respir Crit Care Med. (1994) 149:625–9. doi: 10.1164/ajrccm.149.3.8118629, PMID: 8118629

[22] Allgaier J Lagu T Haessler S Imrey PB Deshpande A Guo N. Risk factors, management, and outcomes of legionella pneumonia in a large, nationally representative sample. Chest. (2021) 159:1782–92. doi: 10.1016/j.chest.2020.12.013, PMID: 33352192

[23] Phin N Parry-Ford F Harrison T Stagg HR Zhang N Kumar K. Epidemiology and clinical management of Legionnaires’ disease. Lancet Infect Dis. (2014) 14:1011–21. doi: 10.1016/S1473-3099(14)70713-3, PMID: 24970283

[24] Wang W Wang Q Xu C Li Z Song Z Zhang Y. Chinese expert consensus on the multidisciplinary management of pneumonitis associated with immune checkpoint inhibitor. Thorac Cancer. (2022) 13:3420–30. doi: 10.1111/1759-7714.14693, PMID: 36268845 PMC9715776

[25] Khunger M Rakshit S Pasupuleti V Hernandez AV Mazzone P Stevenson J. Incidence of pneumonitis with use of programmed death 1 and programmed death-ligand 1 inhibitors in non-small cell lung cancer: A systematic review and meta-analysis of trials. Chest. (2017) 152:271–81. doi: 10.1016/j.chest.2017.04.177, PMID: 28499515

[26] Delaunay M Cadranel J Lusque A Meyer N Gounant V Moro-Sibilot D. Immune-checkpoint inhibitors associated with interstitial lung disease in cancer patients. Eur Respir J. (2017) 50:1700050. doi: 10.1183/13993003.00050-2017, PMID: 28798088

[27] Nishino M Ramaiya NH Awad MM Sholl LM Maattala JA Taibi M. PD-1 inhibitor-related pneumonitis in advanced cancer patients: radiographic patterns and clinical course. Clin Cancer Res. (2016) 22:6051–60. doi: 10.1158/1078-0432.CCR-16-1320, PMID: 27535979 PMC5161686

[28] Ito A Yamamoto Y Ishii Y Okazaki A Ishiura Y Kawagishi Y. Evaluation of a novel urinary antigen test kit for diagnosing Legionella pneumonia. Int J Infect Dis. (2021) 103:42–7. doi: 10.1016/j.ijid.2020.10.106, PMID: 33176204

[29] Mercante JW Caravas JA Ishaq MK Kozak-Muiznieks NA Raphael BH Winchell JM. Genomic heterogeneity differentiates clinical and environmental subgroups of Legionella pneumophila sequence type 1. PLoS One. (2018) 13:e0206110. doi: 10.1371/journal.pone.0206110, PMID: 30335848 PMC6193728

[30] Garcia-Vidal C Sanchez-Rodriguez I Simonetti AF Burgos J Viasus D Martin MT. Levofloxacin versus azithromycin for treating legionella pneumonia: a propensity score analysis. Clin Microbiol infection. (2017) 23:653–8. doi: 10.1016/j.cmi.2017.02.030, PMID: 28267637

[31] Cunha CB Cunha BA. Antimicrobial therapy for legionnaire’s disease: antibiotic stewardship implications. Infect Dis Clinics North America. (2017) 31:179–91. doi: 10.1016/j.idc.2016.10.013, PMID: 28159174

